# Direct and Indirect Costs of Asthma in School-age Children

**Published:** 2004-12-15

**Authors:** Li Yan Wang, Yuna Zhong, Lani Wheeler

**Affiliations:** Surveillance and Evaluation Research Branch, Division of Adolescent and School Health (DASH), National Center for Chronic Disease Prevention and Health Promotion (NCCDPHP), Centers for Disease Control and Prevention (CDC); DASH, NCCDPHP, CDC, Atlanta, Ga; DASH, NCCDPHP, CDC, Annapolis, Md

## Abstract

**Introduction:**

Asthma is one of the most common chronic diseases of childhood and is the most common cause of school absenteeism due to chronic conditions. The objective of this study is to estimate direct and indirect costs of asthma in school-age children.

**Methods:**

Using data from the 1996 Medical Expenditure Panel Survey, we estimated direct medical costs and school absence days among school-age children who had treatment for asthma during 1996. We estimated indirect costs as costs of lost productivity arising from parents’ loss of time from work and lifetime earnings lost due to premature death of children from asthma. All costs were calculated in 2003 dollars.

**Results:**

In 1996, an estimated 2.52 million children aged five to 17 years received treatment for asthma. Direct medical expenditure was $1009.8 million ($401 per child with asthma), including payments for prescribed medicine, hospital inpatient stay, hospital outpatient care, emergency room visits, and office-based visits. Children with treated asthma had a total of 14.5 million school absence days; asthma accounts for 6.3 million school absence days (2.48 days per child with asthma). Parents’ loss of productivity from asthma-related school absence days was $719.1 million ($285 per child with asthma). A total of 211 school-age children died of asthma during 1996, accounting for $264.7 million lifetime earnings lost ($105 per child with asthma). Total economic impact of asthma in school-age children was $1993.6 million ($791 per child with asthma).

**Conclusion:**

The economic impact of asthma on school-age children, families, and society is immense, and more public health efforts to better control asthma in children are needed.

## Introduction

One of the most common chronic disorders in children and adolescents (1), asthma represents a major public health problem of increasing concern in the United States. Between 1980 and 1996, the prevalence of asthma increased by an average of 4.3% per year, from 3.6% to 6.2% among children aged from birth to seven years (2). Since then, asthma prevalence appears to be stable, and in 2001, more than 5 million children aged five to 17 in the United States were reported to have a current diagnosis of asthma (3). Previously published reports (4-8) strongly suggest that asthma not only increases health care use and costs but also places a large burden on affected children and their families. Children with asthma miss out on school, sports, and other childhood activities. Parents or caregivers of children with asthma are affected by missed workdays and decreased job productivity.

In 1992, Weiss et al (4) conducted a study of the asthma-related costs in the U.S. population. They derived estimates of direct medical costs and indirect costs of productivity loss (in 1985 dollars) using data from national surveys, including the National Health Interview Survey (NHIS), the National Hospital Discharge Survey, the National Ambulatory Medical Care Survey, and others. Direct medical expenditures were estimated to be $465 million for children with asthma aged from birth to 17 years, and indirect costs (value of parents' or caregivers' productivity loss associated with school absence days [SADs]) were estimated to be $726 million among school-age children. Because none of the national surveys collects diagnostic and expenditure data, only national expenditure estimates were produced. Although such estimates enable policymakers to understand the economic impact of childhood asthma in the United States, it is necessary to assess the per capita costs for children with asthma to determine the savings or benefits of a successful asthma intervention.

Using the 1987 Medical Expenditure Panel Survey (MEPS), a later study conducted by Lozano et al (6) estimated the per capita health care costs for children with asthma aged one to 17 years, including both asthma-related expenditures only and all care expenditures (both asthma and nonasthma care). For children with asthma aged one to 17 years, the mean asthma-related per capita expenditures totaled $171 per year; the mean all-care per capita expenditures totaled $1129 ($468 for children without asthma). However, those annual cost estimates were based on parent-reported prevalence. Although 8.8% of the study sample was classified as children with asthma (had asthma or wheezing during the past 12 months), 56% reported taking no asthma medication, and 12.3% reported no health care use. To determine the reduction in health costs that would result from an asthma intervention, cost estimates based on treated prevalence or attack prevalence are more appropriate than those using proxy-reported prevalence.

Beginning in 1997, the asthma questions on the NHIS changed the measure of asthma prevalence (2). Now, three measures are used, all restricted to persons with a medical diagnosis of asthma. The first measure is referred to as lifetime asthma prevalence, which includes respondents with a medical diagnosis of asthma at any time in their lives. The second measure identifies persons with a current diagnosis of asthma. The third is a measure of 12-month attack prevalence, which includes the number of persons who had one or more attacks or episodes during the past 12 months.

The objective of this study is to estimate direct and indirect costs of asthma among school-age children using data from the 1996 MEPS. This study is different from previous reports in four aspects: 1) it produces both national estimates and per capita estimates; 2) the 1996 MEPS data are used to derive not only medical cost estimates but also SAD estimates; 3) both medical costs and SADs are estimated based on treated prevalence; and 4) the costs of productivity loss due to premature death among school-age children are estimated. We hope that the results of this study can provide more insights into the economic burden of childhood asthma on society, the individual child, and the family.

## Methods

### General approach

A societal perspective was used to estimate costs of asthma among school-age children. The direct costs of asthma were estimated as asthma-related medical costs. Indirect costs were estimated as costs of lost productivity, including parents' loss of productivity due to asthma-related SADs and loss of productivity due to premature death of children from asthma. Data from the 1996 MEPS were used to derive medical cost and SAD estimates. Published estimates were used for value of lost productivity (9), and data from the National Vital Statistics System were used for asthma mortality among school-age children (2). Both national and per capita estimates were calculated. All costs were in 2003 dollars.

### Data source and data processing

The MEPS is the third in a series of national probability surveys conducted by the Agency for Healthcare Research and Quality (AHRQ)The MEPS is the third in a series of national probability surveys conducted by the Agency for Healthcare Research and Quality (AHRQ) on the financing and use of medical care in the United States. MEPS actually comprises a family of four surveys: 1) a household survey; 2) a survey of medical providers; 3) a survey of health insurance providers; and 4) a survey of nursing home residents. Using the NHIS as its sampling frame, the MEPS household component (MEPS–HC) is designed to provide estimates of health care use, spending, sources of payments, and insurance coverage for the U.S. civilian noninstitutional population. Using an overlapping panel design, self-reports of health care use and spending are collected at the person and household levels through five rounds of in-person interviews that occur during a 30-month period. This yields two full years of data. The MEPS medical provider component (MEPS–MPC) is a survey of medical providers that are directly linked to the respondents in the household survey. The MEPS–MPC is used to replace or to supplement household data to reduce potential bias from relying solely on self-reported data. The MEPS–MPC focuses on medical events and collects information on dates of visit, diagnosis and procedure codes, and charges and payments.

Although MEPS data are generally available from 1996 to 2000, we chose to use 1996 data for this study because only 1996 data have information on SADs. The data used in this article were derived from the 1996 Person-Level Full-Year File (HC-012) and the Person-Level Medical Event Files (HC-001). The 1996 MEPS–HC comprises a sample of 10,597 households and 23,565 individuals. Hispanic households were oversampled at ratios of approximately 2:1; African American households were oversampled at ratios of approximately 1.5:1. The subsample of individuals used in this study consisted of all children aged five to 17 years in 1996 and was further divided into two condition groups: children with asthma and children without asthma. In this study, the definition of children with asthma was further refined to include children who had any type of health care provider visit or prescription medication related to asthma during the year. All other children in the study sample were classified as children without asthma. We identified the children with asthma through diagnosis code 493, according to the International Classification of Diseases, Ninth Revision, Clinical Modification (ICD-9-CM) (10). An event cost associated with an ICD-9 code of 493 was considered an asthma-related medical care cost and was derived from the event files. Using the full-year file, we derived estimates of all-cause medical spending and SADs for each condition group.

Expenditures in the 1996 MEPS are defined as the sum of direct payments for care provided during the year, including out-of-pocket payments and payments by private insurance, Medicare, Medicaid, and other sources. For this analysis, the medical events were classified and enumerated into the following mutually exclusive categories: purchase of prescribed medicines, hospitalizations, emergency room (ER) visits, outpatient hospital visits, office-based visits, other medical equipments, and home health care.

To derive SAD estimates, we had to address two data issues in the 1996 MEPS. First, Round 3 was conducted in 1996 and 1997; some data from Round 3 pertain to 1997. The number of days lost from school in Round 3 that occurred in each calendar year was not ascertained. We developed an algorithm for deciding what portion of the reported SADs occurred in 1996 (the number of days in Round 3 in 1996 divided by the total number of days in Round 3 and then multiplied by the total number of SADs in Round 3). Second, two variables have missing data — the ending date of Round 3 and SADs in Round 3 (34 children with asthma and 583 children without asthma). We used the SAS Multiple Imputation Procedure to impute the missing values for the two variables (11).

### Data analysis

The data were initially processed with SAS software (SAS Institute, Inc, Cary, NC). All estimates produced in this study were weighted to represent the U.S. population. The sampling weights were used to adjust for potential survey response bias. SUDAAN software (Research Triangle Institute, Research Triangle Park, NC) was used to account for the complex sample design in the computation of the final estimates and standard errors for the estimates produced (12). Linear regression analyses were conducted to estimate excess all-cause medical costs and excess SADs for children with asthma compared with children without asthma, controlling for the effects of sociodemographic and access-to-care variables such as age, sex, race, mother's education level, poverty status, and health insurance coverage.

### Calculation of direct and indirect costs

Asthma-related expenditures were considered as the direct costs of asthma; estimates were directly derived from MEPS data. The costs of parents' loss of productivity due to SADs were calculated as the product of SADs associated with asthma and the cost of lost productivity (value of a day lost). The costs of loss of productivity due to premature death were calculated as the product of asthma mortality and the cost of lost productivity (discounted value of future total earnings).

According to a previous published study (9), the value of a lost day was estimated to be $108 (in 2000 dollars) or $115 (in 2003 dollars), which was calculated as the sum of annual earnings and annual household services divided by 365. The same study also reported the present value of future total earnings (including fringe benefits) and of the combination of future earnings and household production. Estimates are reported at each exact age in five-year intervals, beginning with birth. The reported estimates (in 2000 dollars and discounted at 3%) are $1,064,530 for a child aged five years, $1,176,371 for a child aged 10, and $1,290,814 for a child aged 15. In this study, we used the average of the three estimates — $1,254,347 (in 2003 dollars) — as the present value of future total earnings of children who are currently aged five to 17 years.

A recent study by Akinbami and Schoendorf used data from the Mortality Component of the National Vital Statistics System and reported that annual asthma mortality (per million) was 2.7 among children aged five to 10 years and 5.6 among children aged 11 to 17 years during 1995–1996 (2). We used these mortality estimates and the 1996 Census data to calculate the total asthma mortality of children aged five to 17 years as 211.

## Results

As shown in Table 1, of a total of 4786 children aged five to 17 years in our study sample, 248 had asthma-related care during 1996, representing 2,521,537 children in the United States. The annual treated prevalence rate of asthma was 4.9% (2,521,537/51,605,560). Among all of the children with asthma in the nation during 1996, 68% had at least one health care provider visit of some type, and 94% purchased prescriptions for asthma medication and devices. The percentage of children with asthma who used asthma-related care by category of service was 61% for office-based visits, 10% for ER visits, 3% for outpatient visits, and 2% for hospitalization. Sixty-five percent of children with asthma purchased quick-relief medicine for asthma and 36% purchased controller medicine for asthma. Asthma-related medical costs were $1009.8 million ($401 per child) among all school-age children with asthma in the nation. The distribution of asthma-related expenditures shows that 43% of the total expenditure was in prescribed medicine, followed by 36% in outpatient and office-based visits and 21% in hospitalization and ER care.

Table 2 shows the all-cause medical care costs of children with asthma and children without asthma. The all-cause medical costs of children with asthma totaled $2590 million during 1996, with asthma-related care accounting for 39% of the all-care costs. The weighted per capita costs were $1042 for children with asthma and $618 for children without asthma. Compared with a child without asthma, a child with asthma had an excess of $424 in all-cause medical spending. Comparing the cost distribution of children with asthma to children without asthma, children with asthma had a higher proportion of all-cause medical expenditures in prescriptions, office-based visits, and outpatient visits and a lower proportion of expenditures in hospitalization and ER visits.

Table 3 shows the estimates of SADs in children with and without asthma as well as excess SADs of children with asthma compared with children without asthma. Among 2.5 million school-age children with asthma, a total of 14.5 million SADs occurred. On average, a child with asthma missed 2.48 more days of school than a child without asthma (5.81 SADs per child with asthma and 3.33 SADs per child without asthma). The total number of SADs associated with asthma among all of the children with asthma was 6.3 million, accounting for 43% of the total SADs of all of the children with asthma for all illness and injury.

As shown in Table 4, the direct costs of asthma were estimated to be $1009.8 million ($401 per child). The indirect costs of asthma were estimated to be $983.8 million ($390 per child), including $719.1 million ($285 per child) associated with SADs and $264.7 million ($105 per child) associated with premature deaths due to asthma. The total economic impact of asthma in school-age children was $1993.6 million ($791 per child).

## Discussion

This study is the first to produce both national and per capita estimates of direct and indirect costs of children with asthma. Although only 4.9% of U.S. children, or 2.5 million children nationally, had any type of health care provider visit or prescription medication related to asthma during 1996 (parent-reported asthma prevalence was 5.9%), the economic impact of asthma among those children is substantial. The direct costs of asthma were estimated to be $1009.8 million ($401 per child). The indirect costs of asthma were estimated to be $983.8 million ($390 per child), including $719.1 million ($285 per child) associated with SADs and $264.7 million ($105 per child) associated with premature deaths due to asthma. The total economic impact of asthma in school-age children was $1993.6 million ($791 per child).

Unlike most of the previous studies of childhood asthma, this study focuses on children who had any treatment for asthma during 1996. Before 1997, no other national survey collected information to measure treated prevalence or attack prevalence. After the 1997 redesign of the NHIS questionnaire, information to estimate asthma attack prevalence was obtained. The 1996 treated prevalence of asthma derived in this MEPS study (4.9%) is close to the attack prevalence derived from the 1997 NHIS (5.9% among children aged five to 10 years and 6.0% among children aged 11 to 17 years). The annual prevalence of parent-reported asthma in this MEPS study (5.9%) is in the general range of other recent studies, where the prevalence of asthma in 1996 has been reported previously to be 5.5% among individuals aged five to 20 years (2), 7.4% among children aged five to 10 years, and 7.7% among children aged 11 to 17 years (2).

Although two studies by Weiss et al (4,5) have previously estimated direct medical expenditures of asthma in children, their estimates are not comparable to ours because of two major differences. First, they used charges data, which are very different from the payment data we used in this study, to derive cost estimates. Second, they studied costs for all children aged from birth to 17 years, rather than for the school-age children we focused on in this study. Although there is no comparable per capita estimate in the literature to compare with our estimate of asthma-related medical costs, we found that our per capita estimate of asthma-related costs ($401) is very close to our estimate of the excess all-cause medical spending ($424) per child with asthma compared with a child without asthma.

To our knowledge, this is the first study that uses MEPS data to derive SAD estimates for children with asthma. Most early SAD estimates were produced using NHIS data. To compare the results of this MEPS study with those of the most recent NHIS study, one should keep in mind two differences between the studies. First, as noted earlier, the definition of children with asthma is different. In this study, only children who had treatment for asthma were identified as children with asthma. In the NHIS study, the definition of children with asthma was broader: children with asthma did not have to have treatment if their parents believed that they had asthma during the year. Second, the information collected on SADs is different between the two data sets. Although the 1996 MEPS obtained information on the total number of SADs for each child and on the conditions that caused the SADs, it collected no information on the number of SADs associated with a particular condition. Thus, our SAD estimates reflect annual SADs resulting from all conditions. In contrast, the NHIS provided data on the number of SADs resulting from specific conditions (i.e., SADs associated with asthma). Based on the MEPS data, we found that the total number of SADs in 1996 resulting from all conditions was 14.5 million among children with treated asthma (5.75 days per child). On average, a child with asthma had an excess of 2.5 SADs compared with a child without asthma. The study using 1994–1996 NHIS data found that the annual number of SADs associated with asthma was 14 million (3.7 days per child) among children with parent-reported asthma. Although our SAD estimates are different from those of the NHIS study, we found that the proportion of all children with asthma who had asthma-related SADs (49%) is interestingly close to the proportion of all SADs associated with asthma at the individual level (42% = 2.48/5.81). This indicates internal consistency in the MEPS data.

Like many other cost analyses, this study has some clear limitations. The results of this study should be interpreted with some degree of caution. First, as noted earlier, the medical condition data in the MEPS were derived from parents' reports; they may not conform perfectly to diagnoses made by physicians. However, a study by AHRQ staff indicated that, at the three-digit ICD-9 code level, there was agreement between household- and provider-reported conditions in the overwhelming majority of cases (13). In addition, this concern is minimized by the fact that most of the children (94%) who were classified as children with asthma in this study had purchased prescription medicine for asthma. Second, in the MEPS event data file, a health service may occur for multiple reasons; spending associated with specific conditions is not mutually exclusive. However, in this study, we also estimated all medical spending for children with asthma and compared them with children without asthma. Because the estimated per capita asthma-related cost ($401) is very close to the estimated excess cost per child with asthma ($424), it is reasonable to believe that our direct cost estimates are fairly accurate. Third, we did not use asthma medication data as a means of identifying children with asthma; we might have underestimated the treated prevalence as well as the national medical expenditure of children with asthma. There were more probable asthma cases in the 1996 MEPS data. Among children who had neither proxy-reported asthma nor treated asthma in the study sample, 15 probably really had asthma because they purchased albuterol more than once during a year. Fourth, we have significantly underestimated the direct costs of asthma in school-age children, because children with asthma receive a substantial proportion of care in school settings. Because MEPS is limited to expenditures in medical settings, the cost of services provided by the school system, such as nursing care and first-aid care provided by school nurses, school health aids, and school secretaries, were not included in this study. Fifth, the sample size for inpatient, outpatient, and emergency care was small. However, the focus of this study was total medical expenditures across all medical care services, for which we had a sufficient sample size to be confident in both national estimates and per capita estimates.

Even with these limitations, it is reasonable to conclude that the economic impact of asthma to school-age children, families, and society is immense, and more public health efforts are needed to better control asthma in children. Evidence from evaluation studies of asthma intervention programs suggest that asthma among school-age children can be controlled, and significant cost savings in medical care and parents' loss of productivity from asthma-related SADs can be realized, especially when the programs are aimed at children with moderate to severe asthma (14-21). Two economic studies of clinical-based asthma education programs have documented cost savings from $180 per enrolled child with at least persistent asthma to $542 per enrolled child with a history of frequent use of ER services for asthma (14,15). One particularly well-designed randomized controlled trial of an inner-city social-worker–based education program was able to demonstrate cost savings of $2509 per child with one or more hospital visits at baseline, $1050 per child with two or more unscheduled visits at baseline, and $220 per child with more than 50% of days with asthma symptoms (16). Two studies of asthma management programs found a cost savings of $1144 per inpatient child as a result of an inpatient asthma clinical pathway and a cost savings of $1667 per child with one or more hospitalizations or two or more ER visits during a six-month period as a result of a home-based self-management program (17,18). Studies examining the impact of asthma interventions on SADs have also found significant reduction in SADs, including a 1.8-day reduction by a school-based asthma education program, a two-day reduction by a summer asthma camp-based education program for children with moderate to severe asthma, and a 2.5-day reduction by a large-scale population-based asthma management program for asthma patients and their caregivers (19-21).

Based on the medical cost estimates derived in this study, we found that $211.4 million in medical expenditures (21% of the total asthma-related medical expenditures in 1996) are preventable with effective asthma interventions, including $120.8 million in inpatient care and $90.6 million in ER care. In addition, published asthma intervention studies reveal that SADs related to asthma can be reduced by 1.8 to 2.5 days (19-21). Since the cost estimate of parents' loss of productivity derived in this study was based on an additional 2.5 SADs per child with asthma relative to a child without asthma, it is reasonable to believe that more than half of the total indirect costs of parent's loss of productivity ($983.8 million) are preventable with effective interventions. However, to achieve such cost savings, more public health efforts are needed to educate parents and children on the child's condition and medications, the need for follow-up care, and the importance of avoiding known disease triggers. To ensure the success of such effort, education of primary care providers and school staff and efficient collaboration among primary care providers, school health professionals, and health education professionals are essential.

## Figures and Tables

**Table 1:** Asthma-related Medical Care Among U.S. Children Aged Five to 17 Years^a^

	**Number of persons**			**Expenditures^b^**		

**Medical care**	**Unweighted**	**Weighted**	**% of children who used each service (weighted)**	**Per capita weighted expenditure, $ ** **(SE)**	**Weighted expenditure, $ ** **(SE)**	**Weighted expenditure distribution, ** **%**
**Prescription**	230	2,375,040	94	183 (20)	433,761,955 (58,520,972)	43
**Inpatient**	5	55,072	2	2193 (337)	120,768,739 (81,768,373)	12
**Outpatient**	8	76,413	3	635 (377)	48,481,437 (35,537,654)	5
**Emergency**	24	244,170	10	371 (89)	90,592,184 (31,553,044)	9
**Office visit**	153	1,532,728	61	206 (47)	316,158,833 (78,720,894)	31
**Total**	248	2,521,537	NA	401 (54)	1,009,763,148 (147,630,859)	100

a Based on sample size of 4786. Source: 1996 Medical Expenditure Panel Survey. SE = standard error. NA = not applicable.

b Expenditures are presented in 2003 dollars.

**Table 2:** Comparison of All-cause Medical Expenditures of U.S. Children Aged Five to 17 Years With and Without Asthma^a^

	**Children with asthma ** **(N = 2,521,537)**			**Children without asthma ** **(N = 49,084,023)**		

**Medical care**	**Per capita expenditure, ** **$ (SE)**	**National expenditure, ** **$ (SE)**	**Expenditure distribution, ** **%**	**Per capita expenditure, ** **$ (SE)**	**National expenditure, ** **$ (SE)**	**Expenditure distribution, ** **%**
**Prescription**	263 (25)	651,578,798 (78,265,297)	25	146 (21)	3,525,325,923 (510,679,902)	11
**Inpatient**	3328 (819)	284,511,726 (134,082,066)	11	9254 (1999)	9,657,060,475 (2,439,668,298)	29
**Outpatient**	898 (271)	260,576,661 (93,150,742)	10	1068 (189)	3,110,152,939 (598,533,120)	9
**Emergency**	364 (51)	199,370,182 (42,482,913)	8	677 (170)	3,765,138,136 (974,320,040)	11
**Office visit**	435 (66)	954,241,953 (152,081,219)	37	327 (17)	10,774,764,219 (687,815,760)	33
**Other equipment**	558 (372)	239,714,979 (157,341,673)	9	231 (18)	1,538,558,287 (158,934,373)	5
**Home visit**	0	0	0	2,762 (1365)	714,950,437 (365,042,774)	2
**Total expenditure**	1027 (110)	2,589,994,298 (317,235,615)	100	675 (72)	33,085,950,417 (3,598,962,584)	100
**Adjusted per capita expenditure^b^**	1042 (112)	NA	NA	618 (62)	NA	NA

a Source: 1996 Medical Expenditure Panel Survey. All expenditures are weighted and in 2003 dollars. SE = standard error. NA = not applicable.

b Adjusted by age, sex, race, mother’s eduction level, poverty status, and health insurance coverage.

**Table 3:** Estimates of School Absence Days (SADs) Among U.S. Children Aged Five to 17 Years With and Without Asthma^a^

	**Children with asthma**		**Children without asthma**		**Excess SADs per child with asthma compared with a child without asthma**

	**Unweighted** **(n = 248)**	**Weighted** **(n = 2,521,537)**	**Unweighted** **(n = 4538)**	**Weighted** **(n = 49,084,023)**	
**Total SAD**	1440	14,489,164 (1,448,744)	15,454	166,321,913 (6,935,832)	NA
**SAD per child**	5.8	5.75 (0.41)	3.41	3.39 (0.12)	2.36
**Adjusted SAD per child^b^**	NA	5.81 (0.40)	NA	3.33 (0.12)	2.48

a Data are from 1996 Medical Expenditure Panel Survey. All values are numbers (standard errors) unless otherwise indicated. NA = not applicable.

b Adjusted by age, sex, race, mother's education level, poverty status, and health insurance coverage.

**Table 4:** Direct and Indirect Costs of Asthma Among U.S. Children Aged Five to 17 Years (in 2003 Dollars)

	**Direct costs ($)**	**Indirect costs ($)**		**Total costs ($)**

	**Asthma-related medical costs**	**Costs of lost productivity due to asthma-related school absence days**	**Costs of lost productivity due to premature death**	
**National estimate**	1,009,763,148	719,142,352	264,667,217	1,993,572,717
**Per capita estimate**	401	285	105	791
